# Real-Time Dopamine Measurement in Awake Monkeys

**DOI:** 10.1371/journal.pone.0098692

**Published:** 2014-06-12

**Authors:** Erik W. Schluter, Andrew R. Mitz, Joseph F. Cheer, Bruno B. Averbeck

**Affiliations:** 1 Laboratory of Neuropsychology, Division of Intramural Research, National Institute of Mental Health, National Institutes of Health, Bethesda, Maryland, United States of America; 2 Department of Anatomy and Neurobiology, University of Maryland School of Medicine, Baltimore, Maryland, United States of America; University of Chicago, United States of America

## Abstract

Fast-scan cyclic voltammetry (FSCV) is often used to measure real-time dopamine (DA) concentrations in awake, behaving rodents. Extending this technique to work in monkeys would provide a platform for advanced behavioral studies and a primate model for preclinical research. The present study demonstrates the feasibility of DA recordings in two awake monkeys (*Macaca mulatta*) using a mixture of techniques adapted from rodent, primate and brain slice work. We developed a long carbon fiber electrode to operate in the larger primate brain. This electrode was lowered into the striatum each day using a recording chamber and a detachable micromanipulator system. A manipulator also moved one or more tungsten stimulating electrodes into either the nearby striatum or the ventral tegmental area/substantia nigra pars compacta (VTA/SNc). We developed an electrical stimulation controller to reduce artifacts during electrical stimulation. We also introduce a stimulation-based methodology for estimating distances between electrodes in the brain. Dopamine responses within the striatum were evoked by either stimulation of the striatum near the FSCV electrode, or stimulation within the VTA/SNc. Unexpected juice rewards also evoked dopamine responses in the ventral striatum. Thus, we demonstrate that robust dopamine responses can be recorded from awake, behaving primates with FSCV. In addition, we describe how a stimulation technique borrowed from the neuroprosthetics field can activate the distributed monkey midbrain dopamine system in a way that mimics rodent VTA stimulation.

## Introduction

The dopamine (DA) projection of the brain originates from the ventral tegmental area (VTA), substantia nigra pars compacta (SNc) and retrorubral area (RRA) [1], and projects to broad swaths of cortex and basal ganglia [2], [3], with a smaller presence in the cerebellum [4], [5]. Prior to the discovery of this neuromodulatory system, DA had been implicated in the potent reinforcer effect of median forebrain bundle (MFB) electrical stimulation [6]. The effects of rodent MFB stimulation were traced to VTA neurons and DA release through a series of pharmacological, anatomical and electrophysiological studies (see [7]). Compelling models have emerged, emphasizing a putative role for DA in reward prediction [8], [9]. Validating or refuting these models has become a major focus in the study of reward-driven behavior (e.g., [10]).

*In vivo* real-time DA measurement with fast-scan cyclic voltammetry (FSCV) has been developed over the past two decades as a tool for unraveling the role of DA in goal-directed behavior. FSCV is an electrochemical technique for measuring the concentration of an electrolyte in solution using a wide bandwidth two-electrode sweeping voltage clamp. This technique has been extensively developed in rodents [11] where a thin (e.g., 7 µm) strand of carbon fiber is used as the working electrode. It is often used to record DA concentration in the striatum, where norepinephrine (electrochemically indistinguishable from DA), is in sufficiently low concentrations as to not interfere with DA measurements (e.g., [12]). FSCV has important advantages over other *in vivo* techniques for measuring DA concentration because of its ionic specificity, and its temporal and spatial resolutions. Constant potential amperometry is a similar method, but it does not provide information about the ionic species contributing to the current being measured [13]. Optical measurements of DA are not well suited for deep brain use because of the probe size and light dispersion properties of the brain [14]. Positron emission tomography (PET) can be used to estimate DA concentration in voxels of a few cubic centimeters. However, PET has a time course on the order of minutes rather than milliseconds (e.g., [15]). Likewise, although high-performance liquid chromatography (HPLC) offers higher spatial resolution and better selectivity than PET, the sampling time course is typically from 5 to 30 min (e.g., [12]). It is important to note that slower measures of DA capture the tonic levels, typically in the order of 10 s of nM/L, whereas phasic DA release can exceed 1 µM/L (see [11]). FSCV is a high-speed subtraction method; its measurements are localized to the region of the carbon fiber electrode and measurements are taken every 100 ms. Data from FSCV provides localized real-time phasic characteristics that can capture DA release, diffusion and clearance.

Although FSCV has emerged as a staple in rodent research (i.e., [11], [16], [17]), it is not routinely used in primates. Attempts to record FSCV in awake, behaving monkeys [18] or humans [19] have had limited success. From an experimental standpoint, successful FSCV in monkeys would allow one to work in a species with sophisticated behavior and increased anatomical homology with humans. From a clinical standpoint, preclinical success with a monkey model could open the door to on-line, closed-loop monitoring and control of deep brain stimulation (DBS) for treatment of Parkinson's disease (PD), neuropsychiatric and other neurological disorders [20]. However, because of the anatomy of the primate brain, FSCV in monkeys presents challenges not seen in rodent work. Monkeys have a much larger and more diffuse tegmental DA projection to the striatum. Some of the most basic tenets in the rat, like broad activation of this projection with localized VTA stimulation, do not hold in the monkey.

The goal of the present study is to demonstrate the feasibility of using FSCV to measure real-time changes in striatal DA in monkeys. As a prerequisite, we developed a carbon fiber electrode that can reach several cm into the brain while meeting other requirements: easy to reproduce, minimum damage to the brain, accurate targeting, resistance to electrical noise and high sensitivity. In rodents, the primary tool for validating FSCV recordings throughout the striatum is electrical stimulation of VTA, either directly or via the MFB. In monkeys, VTA projects to a small proportion of the ventral striatum [3]. The majority of the midbrain dopamine neurons are spread across ∼6 mm of substantia nigra pars compacta (SNc). Local electrical stimulation anywhere along the VTA/SNc axis leads to restricted activation of DA in the striatum. Finding the restricted area of release in a monkey can be a major challenge in a striatum ∼8 times larger than that of a rat. MFB stimulation does not seem to provide a viable alternative in the monkey. The monkey MFB is apparently not a spatially restricted bundle [21], [22], although groups of MFB fibers can be activated with high currents [23]. Thus, it is not possible to activate the bulk of midbrain DA neurons in a monkey using techniques common in rodents. To address these issues, we employed a mixture of rodent and monkey techniques. This allowed us to survey sites in both the VTA/SNc and the striatum. In addition, we borrowed the technique of local electrical stimulation from brain slice work to expand our ability to survey DA release sites. We were able to demonstrate that FSCV is an effective method for recording DA release from monkey striatum. We also recommend modifications to this hybrid method that will greatly enhance the utility of VTA/SNc stimulation in monkeys.

## Materials and Methods

Unless otherwise stated, the FSCV methods discussed here are similar to those used for rodent recordings by Robinson and Wightman [24].

### Ethics Statement

All experimental procedures were performed in accordance with the National Institutes of Health Guide for the Care and Use of Laboratory Animals and were approved by the Animal Care and Use Committee of the National Institute of Mental Health. Procedures adhered to applicable United States federal and local laws, regulations and standards, including the Animal Welfare Act (AWA 1990) and Regulations (PL 89-544; USDA 1985) and Public Health Service (PHS) Policy (PHS 2002).

Two rhesus monkeys (*Macaca mulatta*) served as subjects in this project, monkey B (9 kg male) and monkey N (7 kg male). Animals were obtained through an approved government source. They were tested for TB and Herpes B twice per year, and received regular dental checks.

Each animal was pair housed with a compatible partner using joining cages in a temperature and automatic lighting controlled (12-hr cycle) animal facility along with other housed conspecifics and no other species. No bedding was in the cage. Environmental enrichment included a toy, most often a chew toy, outside of the cage that could be readily pulled through the bars. Most days, including weekends, animals on study received fruit pieces, often just before or after an experiment. Animals were otherwise fed 12 monkey chow biscuits twice each day at 7 a.m. and 2 p.m. When not on water control, animals had unlimited access to water through a bottle attached to the outside of the cage or a spigot at the rear of the cage. When on fluid control, animals received water every day regardless of performance. Body weight was measured at least once each week and the animal was taken off study if this dropped below 15% of the fully hydrated and fully nourished weight.

Implantation surgeries were conducted in a surgical theater set up specifically for primates. Surgeries were done under a full anesthesia regimen shown effective and safe with non-human primates (ketamine HCl 10–20 mg/kg, i.m. induction followed by isoflurane gas, 1% to 4%, to effect, inhalation) and with sterile technique directed by individuals with appropriate training. Animals were closely monitored prior to, during and after surgery until they could safely sit upright on their own. Postoperative analgesics were administered based on consultation with attending or central facility veterinarian. The regimen most commonly used was ketoprofen (1–2 mg/kg BID x 3 days) followed by ibuprofen (100 mg PO BID x 4 days).

Structural MRI scans were used initially to guide implantation and multiple additional times for health monitoring. For each scan, the animal was sedated or anesthetized, depending upon the duration of the procedure, and monitored throughout the scan and during recovery from anesthesia. The endpoint of the experiment was the first of 1) sufficient data to achieve the stated goal of the experiments, 2) concern that damage from repeated electrode tracks might interfere with recordings (based on MRI scans), or 3) a health issue, including damage from electrodes. Monkey B developed a paralysis after 53 deep electrode tracks. That animal was euthanized using Ketamine sedation followed by deep anesthesia and then transcardial perfusion. Monkey N finished this series of experiments after 32 deep electrode tracks. That animal was subsequently assigned to a different experimental study under the same protocol (animal study protocol number LN-23).

Both animals were naive before the study. They were each gradually adapted to transitions from cages to adjustable primate chairs using a pole-and-collar system and positive (food) reinforcement. Studies took place in either a light and acoustic attenuating chamber designed for non-human primate experiments, or a similar custom-designed inner laboratory room. Animals were adapted to each new environment for days before testing. Likewise, after implantation of a post for head restraint, adaptation to its use was gradual and encouraged with food rewards.

### Implants

**Figure 1:**
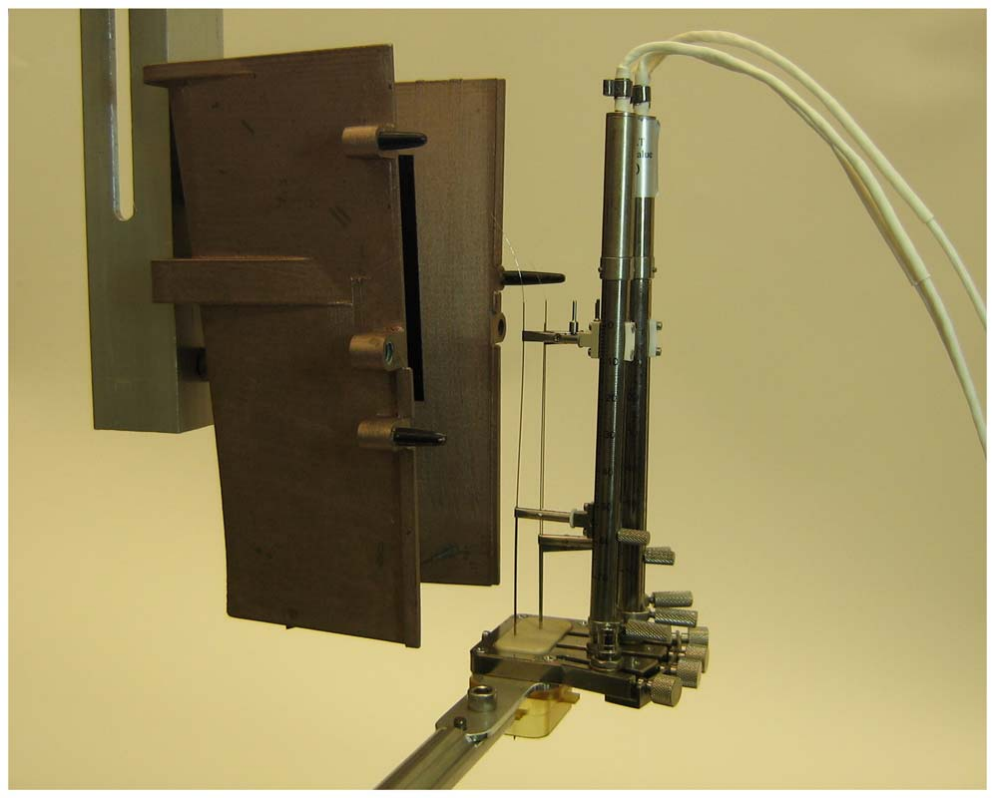
Electrode positioner, shield and chamber arrangement. Electrodes and guide cannulas were back loaded through a 1×1 mm grid and gripped by electrically isolated clamps. Lower clamps could be raised and lowered by hand. Upper clamps were raised and lowered by remotely operated screw-driven stepper motors. The larger component of the Faraday shield has been mounted on a holder for illustration purposes. Normally the smaller component is held fixed (see Fig. 3). Its height and contours snugly fit the recording apparatus, with room near the top to accommodate electrode connections. The arm and plastic recording chamber in this picture are part of the preparation stand. After preparation, the micropositioner and grid are transferred to an equivalent plastic chamber affixed to the monkey's head.

**Figure 2:**
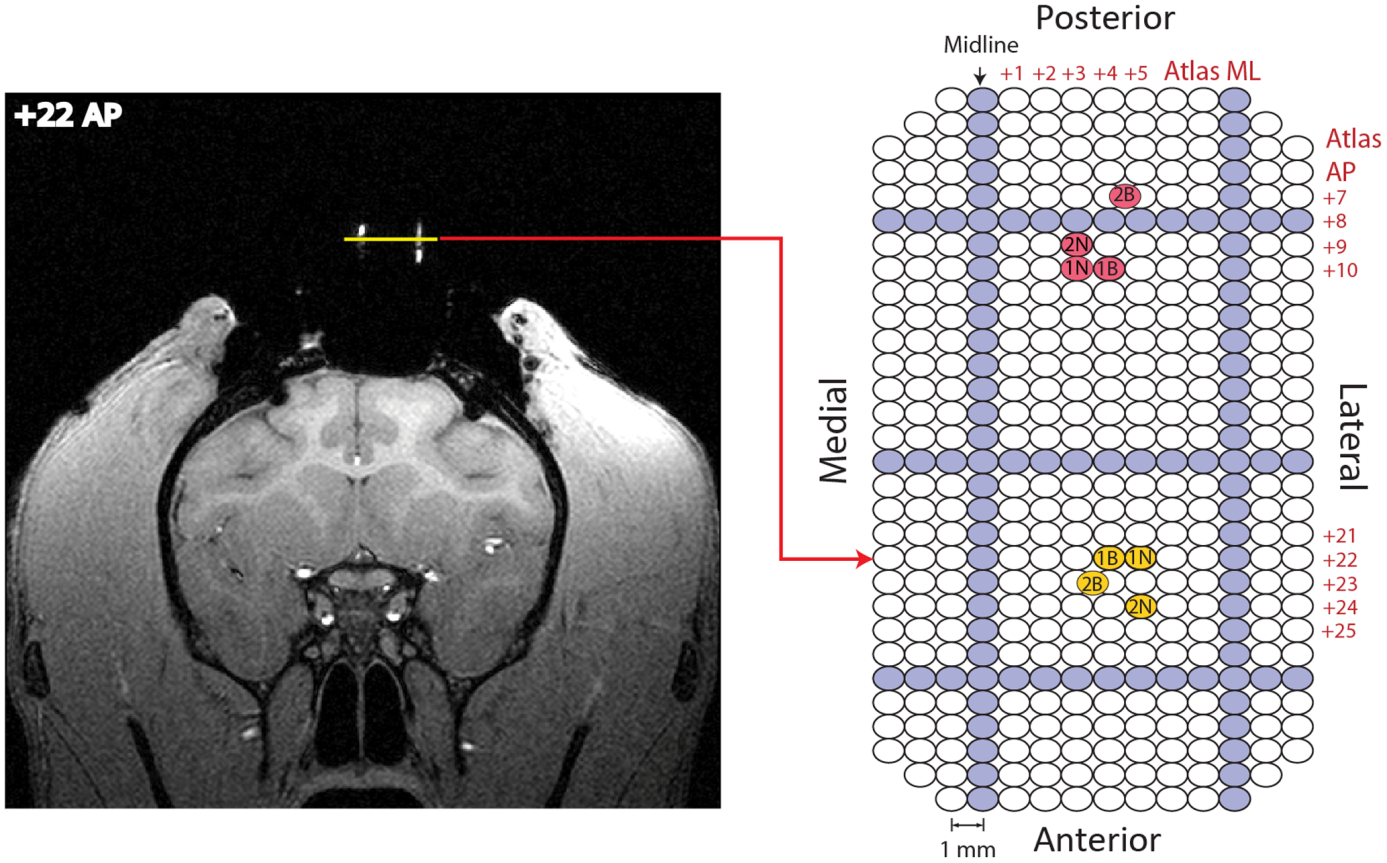
Location of stimulation and recording sites. A grid with 1×1 mm spacing (see Fig. 1) was filled with a contrast agent to align T1-weighted structural MRIs with the coordinate system of the recording chamber. The same grid could be offset by 0.5 mm in the mediolateral (ML) axis, so some penetrations occurred half way between adjacent ML holes in this figure. Left: Frontal MRI section that best matches atlas coordinate AP 22 in monkey B. The horizontal line above the brain is parallel to the plane of the recording chamber. It intersects with two of the filled grid holes, which appear as vertical lines. The distance between the filled grid holes is 8 mm. Right: Map of grid holes used for recording and stimulation. Darker rows indicate holes filled with contrast agent for MRI in monkey B. An arrow shows best AP correspondence with MRI to the left. The AP and ML atlas coordinates of each hole are shown alongside the grid for select grid locations, so that results from both monkeys can be shown here on a common grid map. Example VTA/SNc stimulation (posterior group) and matching FSCV recording (anterior group) sites are plotted on the grid. Matching sites have the same letter/number designation. Letter designates monkey B or N. Table 1 shows the depths of the labeled sites.

Each subject received a custom, plastic 14×30 mm recording chamber (Fig. 1) implanted with the long edge over the midline in a sterile surgery. The rostro-caudal position of the chamber was centered on approximately AP 4, based on the Saleem and Logothetis atlas [25] and a T_1_-weighted structural MRI of each animal prior to surgery. A Titanium head post was positioned posterior to the chamber in the same surgery. After recovery from surgery, a grid matrix was filled with Betadine gel and placed in the chamber just before a second structural MRI (Fig. 2). Candidate grid sites and depth measurements were determined using the free MRIcro software package (http://www.mccauslandcenter.sc.edu/mricro/mricro/mricro.html). Gadolinium was also injected into the brain at defined coordinate positions, and imaged with structural MRI to verify the coordinate system.

### Carbon Fiber Electrode Fabrication and Conditioning

Carbon fiber microelectrodes constructed for rodents (e.g., [11]) are too short for deep structures in larger brains. The specific resistance of carbon fiber used for FSCV is in the order of 1500 µΩ-cm, or about 4.5 KΩ per cm for a 7 µm diameter fiber. This high resistance requires providing a conductive wire electrical connection near the electrode tip. Therefore, we developed a two-stage fused silica design that could be manipulated with a microdrive typically used for single-unit recording. The design is similar to previous electrode designs [26]–[28].

**Figure 3:**
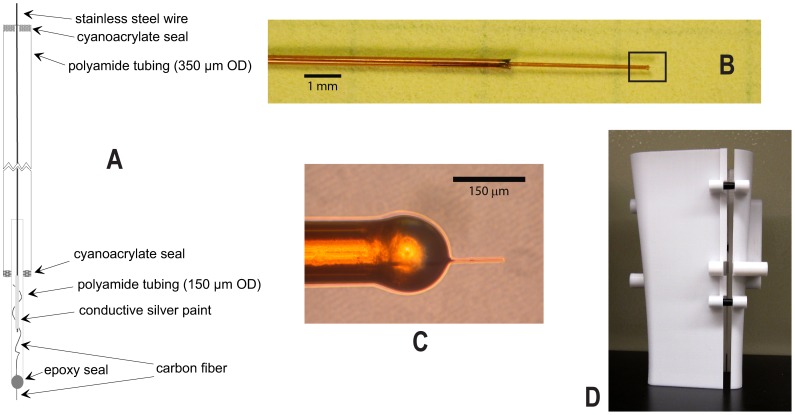
Carbon fiber electrode and printed Faraday shield. (A) Schematic of carbon fiber electrode construction. The 7 µm carbon fiber is electrically connected to a 51 µm (.002 in) diameter stainless steel wire using silver paint. Cyanoacrylate and epoxy provide seals for the two different size polyimide-coated silica capillary tubing. (B) Photograph of the carbon fiber electrode centered near junction of large and small tubes. (C) Expanded region of the black outlined square in tile B is a photomicrograph of exposed carbon fiber and epoxy seal. (D) Two-part Faraday shield. Each side of the clamshell shield was printed on a plastic printer. Plastic alignment pins (in black) and retaining screws (not shown) were added after printing. Base of shield closely fits the dimensions of the microdrive base and chamber (see Fig. 1), providing clearance for the posterior head post. The upper portion is wider to accommodate connections to the microdrive towers. The smaller section is held fixed and the larger section is removable. Photo taken before the application of conductive paint.

The electrode is schematized in Fig. 3A and pictured in Fig. 3B–C. A 4 cm length of carbon fiber, 7 µm diameter (Type C 005722, Tex #795, Goodfellow, Oakdale, PA), is threaded through a 1.5 cm length of polyimide-coated silica capillary tubing (150 µm O.D., 75 µm I.D., Type TSP075150, Polymicro Technologies, Phoenix, AZ, USA). The carbon fiber and silica tube are submerged in a Petri dish of isopropanol during this step. This step is aided by a dissection microscope illuminated from below. Tapping the silica tube against the bottom of the Petri dish dislodges air bubbles in the lumen of the tube and allows the tube to fill completely. The fiber is then pushed into the tube with a cotton-tipped applicator while the tube is stabilized with fine forceps.

Once threaded, the tube with inserted fiber is removed from the isopropanol and mounted with tape on a small platform. The protruding carbon fiber is allowed to hang over the edge of the platform. A scrap piece of tubing is placed underneath the opposite end of the tube to lift it from the platform. The fiber protruding out the elevated end can then be pulled through the tube. The fiber is glued at one end of the silica tube with two-part epoxy gel (Devcon 14240, ITW Devcon, Danvers, MA) using a stainless steel wire to apply the mixed gel. Care is necessary to limit the epoxy contact with the fiber near the entry point of the tube. The fiber is then pulled slightly into the tube as the epoxy is applied, so that some of the epoxy joint is inside the tubing and the epoxy outside the tube tapers smoothly (Fig. 3C). The epoxy cures in about two hours. After curing, the assembly is taped to a flat surface. The exposed carbon fiber on the glued end is cut to a length between 125–150 µm with iridectomy scissors. This completes the first stage of the assembly.

A second stage “shaft” is necessary because: 1) the overall length of the electrode needs to be about 10 cm, 2) the small capillary tubing is not stiff enough to track a straight trajectory in the brain, and 3) the carbon fiber is a poor conductor. The second stage is constructed using larger capillary tubing with internal stainless steel wire. This larger polyimide-coated silica capillary tubing (350 µm O.D., 180 µm I.D., Type TSP180350, Polymicro Technologies, Phoenix, AZ) is cut to length as necessary (typically 95 mm). A 0.051 mm diameter stainless steel wire (part number 792600, 0.002 in, half-hard, 155–185 kPSI, A–M Systems, Sequim, WA) is threaded through the larger capillary tubing. The wire is cut so about 2 cm extends beyond each end. The carbon fiber assembly is taped flat onto an opaque surface under the dissecting microscope; here, the assembly is illuminated from above. The stainless steel wire is coated on one end with conductive silver paint (05002-AB, SPI Supplies, West Chester, PA). The next steps must be done quickly while the silver paint is still wet. The painted stainless steel wire is inserted into the back of the carbon fiber/silica tube assembly, which pushes the carbon fiber deep into the assembly. Care must be taken to ensure the carbon fiber does not fold too sharply and break. The large tube is slipped over the small tube, leaving about 8–10 mm of the small tube protruding from the large one. The two capillary tubes are sealed together with cyanoacrylate (KG585, Krazy Glue, Columbus, OH). The stainless steel wire extending from the back of the large capillary tubing is also sealed with cyanoacrylate and trimmed to about 2 cm. This wire provides an electrical contact for the voltammetry head stage. The full assembly is allowed to sit overnight before use.

Carbon fiber microelectrodes were cycled at 60 Hz for approximately 30 min in aCSF solution just prior to use to condition the carbon surface and thus raise sensitivity for recording stability [29]. We monitored stability by quantifying baseline shifts during every 2-min voltammetry recording, both in the flow cell and *in vivo*. Electrodes that could not maintain a stable baseline (less than 5 nA change in any 10 s epoch) in the flow cell were rejected. Those that could not maintain a stable baseline in the brain were removed and recordings were usually terminated for that day. Background noise of the electrode was an equally important factor, because it established the minimum resolution of the background-subtracted DA measurements. Electrodes with subtracted noise levels above 0.5 nA were rejected. When excessive electrode noise developed during an experiment, recordings were terminated. Electrodes were fabricated in batches of 10 to 20 electrodes. About half met criteria for use after flow cell testing. The shelf life of an electrode was from 3 to 7 days.

### Reference Electrode Fabrication

Ag/AgCl electrodes were used to reference the voltammetry signals. A length of high purity 30 gauge silver wire (AG10X, Sigmund Cohn/Medwire, Mt. Vernon, NY) was cut and electrolytically coated with chloride using a regulated power supply set to 9 V DC. The silver wire was connected to the positive terminal via a clip-lead and dipped 1 to 2 cm deep into 0.1 N HCl solution for 10 s. A submerged stainless steel ground wire served as the return lead for the 9 V power source. The coated silver wire was threaded through a 95 mm length of large silica capillary tubing, as used in the carbon fiber electrode fabrication. The coated end was extended 7 mm from one end of the tube and glued in place with cyanoacrylate. The other end was also sealed with cyanoacrylate and trimmed to a convenient length for electrical connection.

### Electrical Stimulation

Electrical stimulation was used to produce large, time-locked DA release. Stimulation-evoked DA release provides a strong DA signal to test electrodes *in vivo*, helps locate regions of the striatum where DA signals are present, and provides a tool to monitor the effects of pharmacological agents in awake primates.

We tested two different approaches to electrical stimulation: local stimulation near the carbon fiber electrode in the striatum and VTA/SNc stimulation aimed at the cell bodies or perhaps axons of midbrain DA neurons. VTA/SNc stimulation was effective in 3 of 12 attempts from monkey N and in 2 out of 20 attempts from monkey B. Local stimulation was effective in 10 out of 14 attempts in monkey N. In either case, Epoxylite coated 250 µm diameter sharpened Tungsten electrodes (FHC Inc., Bowdoin, ME) were used both for single unit recording and monopolar electrical stimulation. For stimulation, insulation near the tip of stock electrodes was removed with a sharp scalpel to increase the surface area. We selected 10–90 kΩ impedance (Z, measured at 1 KHz in saline using a sinusoidal waveform) electrodes for local stimulation and Z = 50–140 kΩ electrodes for VTA/SNc stimulation. The differences in these ranges reflect the evolution of our methods more than any systematic choice based on the region being stimulated. Low impedance electrodes are appropriate for the currents used in this study. The electrodes with Z near 100 kΩ have ∼1000 µm^2^ surface area and relatively blunt tips. These electrode characteristics are preferred for repeated electrical stimulation deep in the brain [30]. The distribution of current spread beyond ∼50 µm from the electrode tip resembles that of a point source [30], so it is easier to make estimates of current spread compared to bipolar electrodes. Bipolar electrodes are, by necessity, larger. We chose to avoid the larger electrodes because our experiment used repeated entry into different parts of the VTA/SNc and striatum. Bipolar electrodes would have caused substantially more damage.

We kept most stimulation currents below 400 µA and all stimulation was symmetric biphasic to minimize the negative effects of charge density and net charge transfer. These characteristics are in line with other studies of stimulation evoked DA release (e.g., [10], [31]). When higher currents were tested, they never exceeded 3 times the threshold for a nearby response. Given the long-term, e.g., hours, of stable evoked DA responses we observed in this study and our extensive experience using the same stimulation method mapping cortical motor areas [22], [32], it is evident that our electrodes and stimulation parameters are effective and sufficiently benign.

The timing of electrical stimulation was controlled by a custom-built circuit that generated the number and frequency of stimulation pulses (Appendix S1). It tracked the FSCV sweeps and avoided these sweeps to assure electrical stimulation did not produce artifacts in the voltammograms. Optionally, it was often programmed to interfere with just the first stimulus pulse to provide a timing marker on the voltammogram. Once the stimulation timing pulses were generated, these pulses were delivered to a Master-8-vp (AMPI, Jerusalem, Israel) to provide cathode-first biphasic rectangular pulses of varying amplitudes in the ±2 V range. In the last step, these biphasic voltage pulses drove a biphasic linear isolation unit (LSIU-01, Cygnus Technology, Delaware Water Gap, PA) connected to the stimulation electrode and referenced to the same electrode's stainless steel guide cannula. The isolation unit has an electrode shorting input to assure no current can flow between stimulus trains. The shorting circuit was managed by the microprocessor-based controller. The internal calibration of the Master-8-vp output voltage range was readjusted to match the isolation unit range (±2 V), which permitted finer resolution of the delivered current. Stimulation was delivered as symmetric biphasic pulses, 500 µs/phase, cathodal first. Stimulation trains were delivered at 120 PPS and lasted for approximately 1 s, unless otherwise noted. Stimulation was delivered starting 20 s into each 40 s voltammetry recording. Unless otherwise noted, a minimum of 5 min separated each stimulation pulse train.

### Microstimulation

On a few occasions, we observed and noted evoked movements in response to microstimulation in the midbrain. For these tracks, 100 ms pulse trains were delivered through tungsten electrodes (Z = 15 kΩ) at 300 PPS. Stimulation was delivered as symmetric biphasic pulses, 500 µs/phase, cathodal first. There was no specific wait time imposed between these stimulation trains. All stimulation was delivered using the guide cannula of the stimulation electrode as the reference electrode.

### Voltammetry

#### Electrode selection

**Figure 4:**
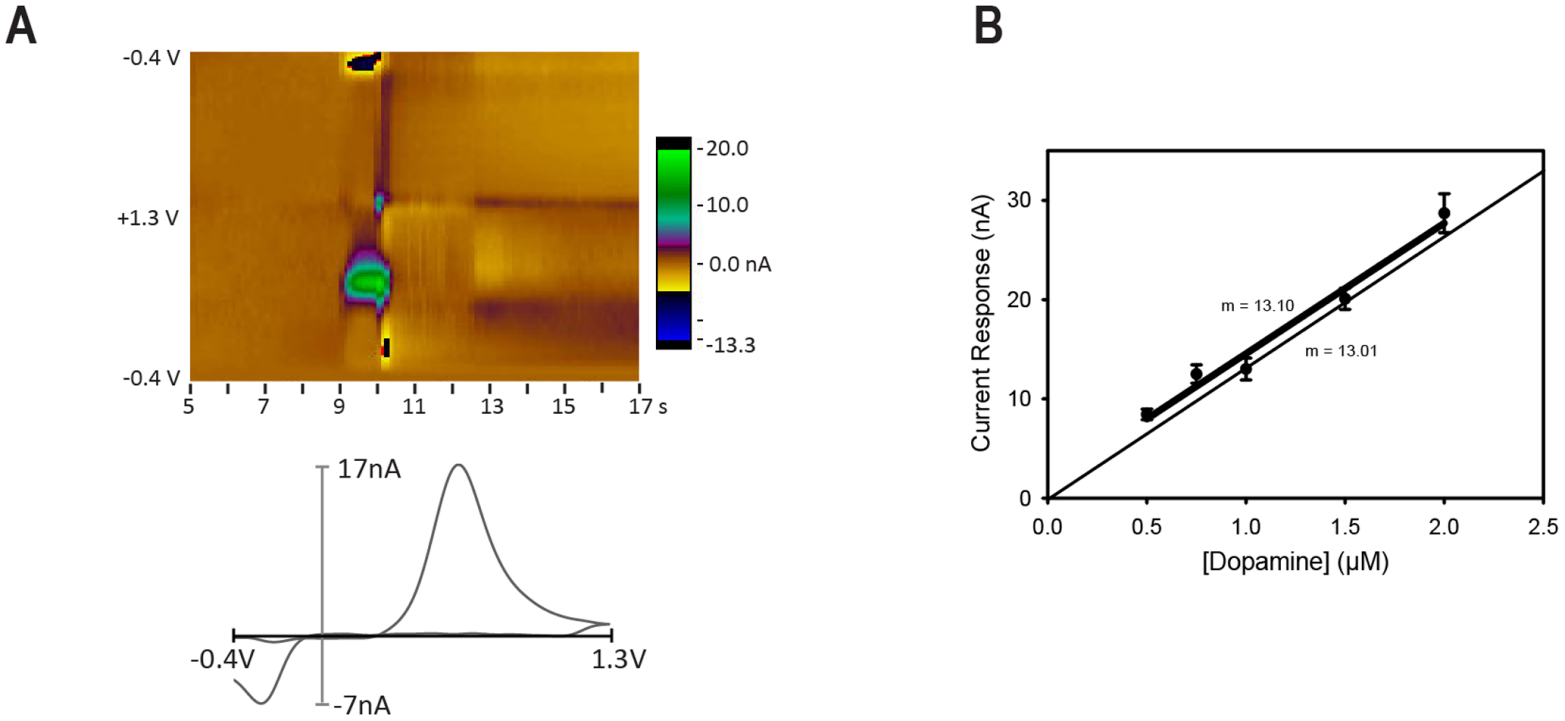
Electrode selection. Electrodes were selected for low noise, stability and reasonable sensitivity. Just prior to recording, available electrodes were tested by injecting a single 1.0 µM, 1–5 s bolus of DA at 1.5 ml/min in a flow-cell apparatus. (A) Pseudo-color plot and corresponding voltammogram response to single bolus at 1.0 µM. The measured peak value is 16.9 nA at 0.62 V. (B) Thick line: complete calibration of another electrode calculated by averaging 4–5 samples of each concentration from the series 0.5, 0.75, 1.0, 1.5 and 2.0 µM (sensitivity  = 13.1 nA/µM). Error bars are SEM. Thin line: simplified calibration using only the values collected at 1 µM and the origin (sensitivity  = 13.0 nA/µM).

Voltammetry was carried out using a version of the Tar Heel CV recording system developed at the University of North Carolina, and updated and supplied by Dr. Scott Ng-Evans (Seattle, WA). A matching system was used for flow-cell testing and electrode selection. Flow cell components were provided by the same vendor. This system uses a syringe infusion pump (Model 22, Harvard Apparatus, Holliston, MA) to deliver a constant flow of 1.5 ml/min through a 250 µL sample loop, switched in-line by a 2-way injection valve (V-1451-DC, Upchurch/IDEX, Oak Harbor, WA). Carbon fiber electrodes were fabricated with target flow-cell sensitivity between 10 and 25 nA/µM (Fig. 4A).

The goal of FSCV is to measure the concentration of a chemical species in the brain by measuring current flow during oxidation. The voltage between a working electrode and suitable reference is swept through the peak oxidation potential, and then swept back through the reduction potential, for the chemical species being measured [11]. During these sweeps, the current flow through the working electrode is converted to a voltage with a high-gain gyrator circuit, and then digitized. When subtracted from background current, the current measured during a sweep provides a signature for the chemical species and an estimate of the change in concentration of that species in solution. The rising voltage sweep passes through the peak oxidation potential of DA (+0.6 V versus Ag/AgCl reference), and current differences of about 1 to 50 nA reflect interstitial concentration changes of DA. The very small surface area of the working electrode permits rapid measurements in a small volume of tissue and produces small oxidation/reduction currents.

Calibration of electrode sensitivity (nA/µM) is necessary to estimate DA concentration. Calibration requires bathing the electrode in known concentrations of the electrolyte in a flow cell. These are idealized conditions, however. Voltage scanning parameters [11], electrode conditioning [33] and environmental factors limit in vivo accuracy based upon flow cell electrode calibration. Environmental factors affecting current response to intracerebral DA include local calcium (Ca^2+^) and magnesium (Mg^2+^) concentrations [34], interference by pH and O_2_ changes [18], fouling of the carbon fiber surface by extracellular metabolites [35] and the difference between flow-cell temperatures and body temperatures [36].

The flow cell was used to select candidate electrodes. On each recording day, the sensitivity of each available electrode was estimated by a single flow cell measurement at 1.0 µM DA concentration. An example is shown in Fig. 4A. Only electrodes that produced stable recordings, low noise, and current values nearest to 20 nA, were selected for use.

To correlate this single measurement with electrode sensitivity, we ran a complete calibration on a single sample electrode using 5 different test concentrations (0.5 µM, 0.75 µM, 1.0 µM, 1.5 µM, 2.0 µM) of DA solution (Fig. 4B). All test solutions were prepared from a 2.0 mM stock solution of DA mixed the day of calibration. First, a 20.0 µM solution was made from the 2.0 mM stock, then a 2.0 µM solution was created from the 20.0 µM solution. Test solutions were mixed in amber vials wrapped in foil to prevent light exposure and kept over ice prior to flow cell loading. Electrode calibration began with the smallest test concentration (0.5 µM) followed by increasing concentrations. The injection circuit was flushed with DI water and cleared with air between concentrations. Recording files were 20 s in duration with a 1–5 s DA injection during the recording. Each concentration was tested 4–5 times. The average peak current at the oxidation potential of DA (near +0.6 V) was plotted at each concentration (Fig. 4B). The slope of the regression line is 13.1 nA/µM, which is taken to be the actual sensitivity of the electrode in aCSF. The first value at each concentration was higher than subsequent values. At 1.0 µM DA concentration, the first value was 17.4 nA. We note that taking an average of 5 measurements at 1.0 µM provides a substantially better estimate (Fig. 4B). Thus, we presume that relying on a single flow cell test tends to overestimate the electrode sensitivity in aCSF.

#### Shielding and microdrive system

Faraday shielding for single unit and voltammetry recording was provided by a custom two-part plastic encasement printed on a Fortus 360 mc 3D plastic printer (Stratasys, Eden Prairie, MN), using ABS-M30 material (Figs. 1, 3D). All plastic surfaces were coated with CuPro-Cote conductive paint (Less EMF Inc, Latham, NY) using a paintbrush. The encasement was air dried for 48 hours before use. The voltammetry, reference and recording/stimulation electrodes were each mounted on a stepper motor-driven multi-electrode microdrive (NAN-S4 with custom base, NAN Instruments, Nazareth, Israel), and each was guided by a sharpened 22 gauge standard-wall stainless steel cannula (Fig. 1). Each microdrive tower provides manual movement of a guide cannula and precision movement of an electrode along the same axis. The shield was designed to provide a snug fit to the base of the recording chamber and a loose fit near the electrode wire connections. The shield can be opened and closed without disturbing the electrical connections. The shield height matched the stepper motor tower heights. The posterior, smaller component of the encasement was shaped to avoid the Titanium head post. To determine the shape, a model was first molded using many layers of aluminum foil with all the recording components mounted on the subject's head. The 3D aluminum foil structure was then cut along the long axis for removal and rejoined with thermal glue. Additional glue helped stabilize the model. The final design was drawn as a straight-line segment approximation of the model. The split between the two shield components was located for maximum access to electrodes when the larger component was removed.

Electrode mounting was performed prior to recordings on a support arm (Fig. 1). Cannulae were inserted into a grid array of holes and a microdrive tower was adjusted to align with each cannula. The grid could be inserted in two different orientations, providing a 0.5 mm medial-lateral offset between the orientations. After insertion, cannulae were clamped to a lower, manual positioner and remained electrically isolated from the tower and base. Electrodes, one of each type, were inserted into each cannula and clamped to the upper, stepper motor positioner, also electrically isolated from the other components. The electrodes were then carefully aligned to the lower margin of a 30 mm×14 mm grid array of drilled guide holes using the positioning motors. After loading and alignment, the assembly was transferred to the recording chamber on the monkey. The guide cannulas were carefully lowered through the dura by hand, each to a predetermined depth. The reference electrode cannula was placed just below the dura. The other two cannulas were lowered to within 3–5 mm above the electrode final target positions. The Faraday shield was closed as the electrodes were automatically lowered to the bottom of their respective guide cannulas. The reference electrode was inserted into the brain first, followed by the carbon fiber electrode. The stimulation electrode was then lowered to its starting position in the brain. All electrodes were lowered at a maximum rate of 0.1 mm/s while still in the guide tube, and 0.01 mm/s in the brain until reaching the initial recording and stimulation sites. Once the electrodes were in position, the carbon fiber electrode was cycled at 10 Hz for up to 45 min. Electrodes were then moved at 0.001 mm/s between recording and stimulation sites.

During local stimulation, voltammetry recordings were often made after electrode movement, but before stimulation, to assess the stability and noise characteristics of the carbon fiber electrode. The stimulation electrode was advanced in 0.1 mm steps and stimulation was tested at every depth until finding a strong response without an overwhelming artifact. Stimulus trains were always separated by at least 5 min following a DA response. After choosing a final position for the electrode, the time of the first stimulation train at this depth was assigned time  = 0 for the purpose of testing response stability.

#### Recording sessions

During setup, the carbon fiber electrode microdrive tower was positioned over a grid hole above the striatum. When used for local stimulation, the stimulation electrode tower was positioned over a grid hole 1 mm posterior to the carbon fiber electrode in the same ML plane. Alternatively, for VTA/SNc stimulation, the stimulation tower was placed over a grid hole positioned above the VTA/SNc. The reference electrode tower was placed above the primary motor cortex, 7–10 mm lateral to the midline. Stimulation and recording tracks were chosen based on whether or not the track had been explored previously, whether DA had been found along that track, or whether multiple electrodes broke along the track. We chose, initially, to explore most of the available tracks throughout the ventral striatum before returning to previously explored tracks, even though we were successful in evoking DA from previously explored tracks. When we were able to record DA release and did not move the stimulation electrode, the stimulation-evoked response remained consistent throughout the recording session. During these recording sessions, stimulation evoked DA was recorded every 5 min until the end of the experimental protocol.

The stimulation controller (Appendix S1) could be programmed to deliver single microstimulation stimuli during the next FSCV sweep following a button press. The size of the “artifact” recorded by the voltammetry system provides a relative measure of distance between the stimulation and carbon fiber electrodes. We used these artifacts to evaluate the accuracy of our electrode positioning measurements by estimating the distance between electrodes as a function of normalized current strength. Current measurements as a function of electrode position were fitted to: 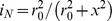
where 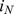 is normalized current,
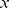
is the distance between electrodes and
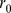
is the nearest approach. Derivation and application of this approach is provided in Appendix S2. Errors in nearest distance measurements and in depth measurements were estimated separately after fitting the equation. This technique provides measurements separate from the microdrive system, which is subject to the flexible nature of the carbon fiber electrode and the long distances traveled by both electrodes.

With each new animal, single unit recordings, usually in the first week of recording, helped locate deep structures in the region of the VTA and substantia nigra. Unit recordings were made with one of two Tucker-Davis Technologies RZ series recording systems (RZ2 and RZ5, Tucker-Davis Technologies, Alachua, FL). A custom remote-switching head stage permitted recording and stimulation through a single electrode in monkey B.

## Results

### FSCV Response to VTA/SNc Stimulation

**Figure 5:**
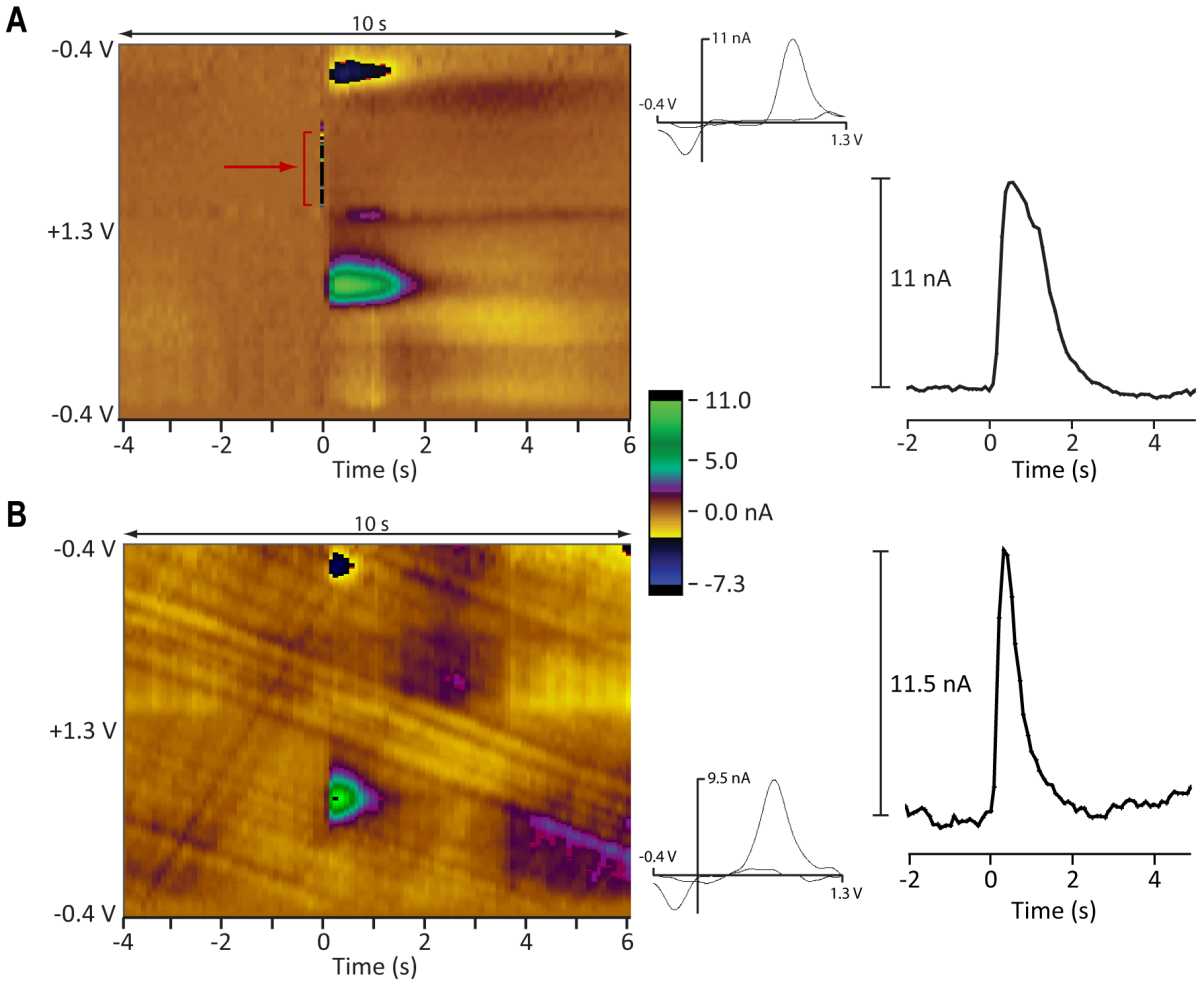
VTA/SNc stimulation-evoked response in two subjects. VTA/SNc stimulation-evoked dopamine response recorded from the ventral striatum of two monkeys. (A) Monkey N. Stimulation parameters: 400 µA, 120 PPS, 1 s train. Left: background subtracted color plot corresponding to the pair of sites designated 2N in Fig. 2 and Table 1. DA release follows rapidly after stimulation at time 0. The red arrow and bracket point to the intentional stimulation artifact that marks onset of stimulation. Right: Small inset graph shows the voltammogram at maximum DA release. Larger plot shows the current response at peak oxidation potential. (B) Monkey B. Stimulation parameters: 200 µA, 120 PPS, 2 s train. Left: background subtracted color plot corresponding to the pair of sites 1B in Fig.2 and Table 1. DA release following stimulation at time 0. Right: Same plots as in A. Vertical color bar shows pseudocolor calibration for both color plots.

We carried out experiments in which a stimulation electrode was introduced into the VTA/SNc and a carbon fiber electrode was introduced into the ventral striatum. Electrode locations were verified with MRI (see Implants section). Example stimulation and recording sites from both monkeys are shown in Fig. 2 with atlas coordinates and peak evoked currents in Table 1. Fig. 5A shows an example of evoked DA release in the striatum of monkey N when the stimulation electrode was in the ventromedial aspect of the SNc (Fig. 2 and Table 1 grid position 1N). The red arrow in the color plot shows where the first pulse of the stimulator intentionally interfered with the first voltammetry scan cycle of the stimulation period, providing a marker 20 s into the recording period (see Appendix S1). The maximum stimulation evoked response in this example was about 11 nA, using a 400 µA stimulation current. DA release was rapid (latency <100 ms) and the response persisted throughout the entire 1 s stimulation train period. We also evoked dopamine release by stimulating DA neuron cell bodies in monkey B (Fig. 5B, grid position 1B in Fig. 2 and Table 1) and, in this case, the largest peak response was over 11 nA. VTA/SNc evoked dopamine oxidation peaks in the brain were higher than oxidation peaks recorded in the flow cell, typically by about 160 mV.

**Table 1:** VTA/SNc evoked dopamine responses.

	Stimulation			FSCV Recording			
Site	AP	ML	EBZ	AP	ML	EBZ	Current
1N	10.0	3.0	9.0	22.0	5.0	17.0	11.0 nA
2N	9.0	3.0	8.0	24.0	5.0	17.5	1.5 nA
1B	10.0	4.0	11.4	22.0	4.0	16.1	11.5 nA
2B	7.0	4.5	9.5	23.0	3.5	9.5	10.0 nA

Site designations match those in Fig. 2. AP, ML and EBZ (ear bar zero) are atlas coordinates [25]. Current is the largest dopamine current evoked at the site.

**Figure 6:**
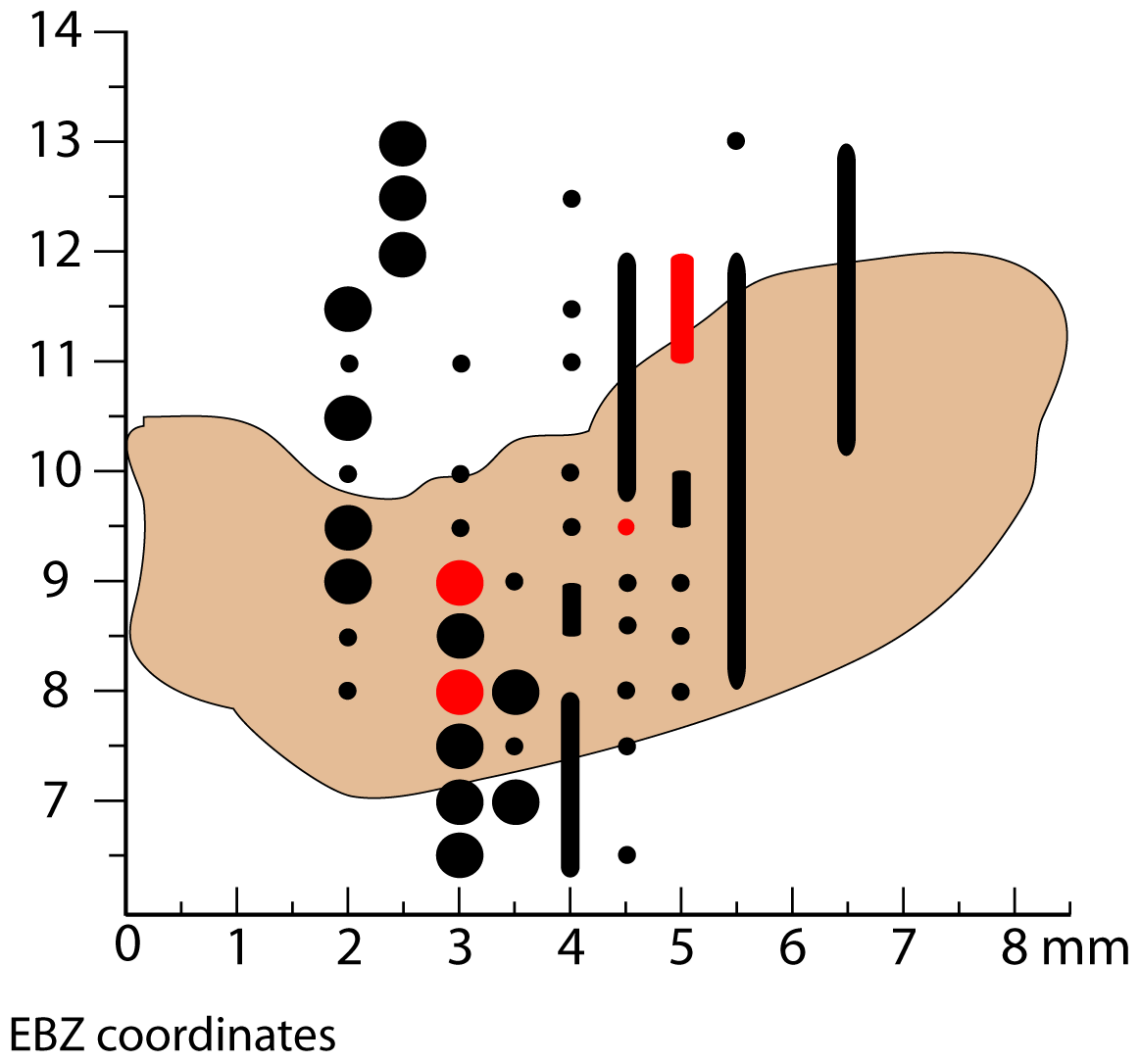
Distribution of stimulation sites in the VTA/SN. Outline of VTA/SN from all AP levels projected onto a single AP plane. The origins of the remaining axes are relative to ear bar zero (EBZ, see description in [25]). Black circles represent all stimulation sites where no dopamine release was observed. Red circles represent all stimulation sites where dopamine release was recorded. Small circles indicate stimulation currents ≤300 µA. Large circles indicate application of a >300 µA, typically 400 µA current. The vertical columns represent a series of stimulation sites along the rostral-caudal axis separated by less than 250 µm. In these columns, color and size codes remain the same.

Our stimulation covered much of the SNc that projects to the caudate and ventral striatum and a portion of the VTA (Fig. 6). The medial most part of VTA was inaccessible with our microdrive arrangement because it would have risked damage to the superior sagittal sinus. This part of the VTA projects to the most medial and most ventral regions of the striatum [3]. The most ventral regions of the striatum were explored sparingly during the stimulation experiments to limit damage to the remainder of the striatum.

**Figure 7:**
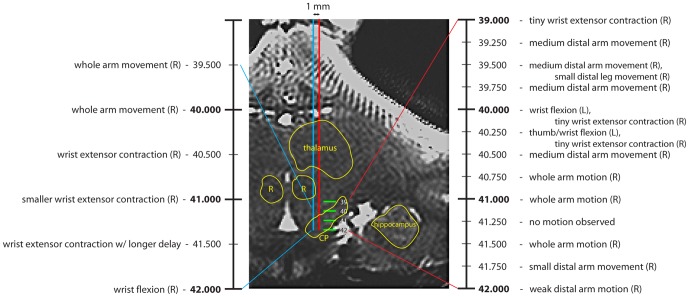
Microstimulation mapping of movements evoked near VTA/SNc. Microstimulation was used to map observed limb movements evoked along two tracks in Monkey N. 100 µA was used for the more medial track (ML 4.5), 50 µA was used 1 mm more laterally (ML 5.5) in the same AP (+9) plane. Stimulus trains were 100 ms at 300 PPS. Actual depths (below cortical surface) are denoted on the scan image. Expanded scales on the left and right indicate observed movements. All movements were brief and time-locked to the stimulation. Anatomical landmarks are outlined on the image (CP – cerebral peduncle; R – red nucleus).

Larger stimulation currents may have allowed us to evoke DA release more consistently in the striatum. However, it was rarely possible to employ large (>400 µA) currents when stimulating in VTA or SNc. Electrical stimulation within the VTA/SNc can evoke movements from the nearby magnocellular division of the red nucleus (RN) and the cerebral peduncles. The rostral tip of RN is just lateral to VTA. Further, SNc is sandwiched by RN dorsally and the cerebral peduncle ventrally [25]. Unwanted evoked movements mechanically disturb the carbon fiber electrode and thereby produce voltammetry recording artifacts. We observed stimulus evoked movement latencies in the voltammetry recordings as brief as 200 ms (two voltammetry cycles). We decided to map the somatotopy of stimulation-evoked movement along two tracks to verify that current spread to known motor structures was the source of evoked movements. Fig. 7 shows the two electrode tracks where microstimulation evoked movements using short stimulus trains (50 ms duration, 30 pulses) and relatively low currents (50 µA). The results from these two tracks suggest that large movement artifacts were caused by current spread to the RN [37] or to corticospinal fibers passing through the cerebral peduncles [38], but not to thalamic sites; thalamic stimulation sites at this AP level did not produce movements or movement artifacts.

### FSCV Response to Local Stimulation

**Figure 8:**
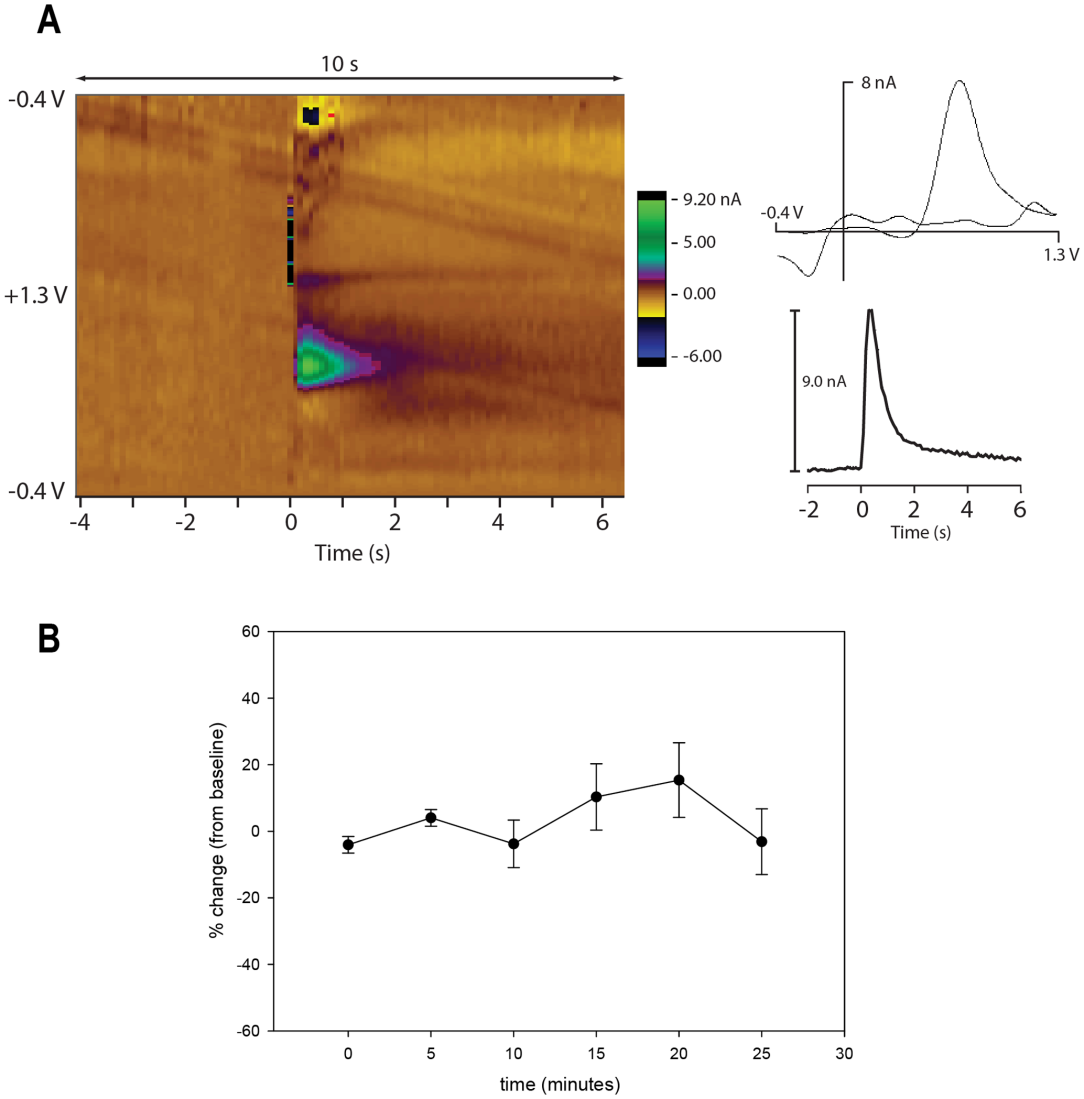
DA recordings during local electrical stimulation. Local electrical stimulation and DA recording together in ventral striatum of Monkey N. (A) Example of one stimulus train: 50 µA, 120 Hz, 1 s duration. Carbon fiber electrode was at AP 24, ML 4.5, EBZ 21.2 (relative to ear bar zero, see [25]). Stimulation electrode was 1 mm posterior and 0.2 mm dorsal. Left: background subtracted color plot showing example of DA release following stimulation at time 0 (marked by stimulus artifact). Right: Corresponding cyclic voltammogram and plot of the carbon fiber current at peak oxidation potential. (B) Stability of DA signal using measurements from 6 different recording days. Electrode positions and stimulation currents were held fixed while periodically testing the DA response. Average is plotted as percent change from baseline DA signal ± SEM. Baseline was calculated as the average of first two samples each day. Samples were collected every 5 min for 25 min (4 days) and 20 min (2 days).

We also carried out local stimulation in the striatum, near the carbon-fiber electrode (<1 mm). This approach is commonly used in rodent brain slice preparations of striatal tissue. The distance between the stimulation electrode and the carbon fiber electrode is generally 100 to 400 µm in brain slice experiments [10], [39], [40]. The mechanism of DA release in striatum is consistent with axonal depolarization by the stimulation current [40]. These studies use bipolar electrodes and stimulus currents in the hundreds of microamperes. In the present study, monopolar stimulation was used deep in the brain to prevent the additional damage caused by inserting a larger bipolar electrode each day. Fig. 8A shows a representative phasic DA release event in the ventral striatum evoked by local electrical stimulation at 50 µA. We applied local stimulation in monkey N at sites in both the ventral striatum and more dorsal-laterally in the putamen.

Voltammetry recordings made after electrode movement, but before stimulation, did not show any transient DA release attributable to movement of the stimulation electrode. However, we did not attempt to measure changes in the background level of DA that may have resulted from movement of the stimulation electrode.

In 6 of the days that we were able to evoke release, we tested the stability of the DA recording for at least 20 min. On those days, we continued to record stimulation-evoked DA responses every 5 min over a 20 min period. On 4 of 6 days testing was continued for at least 5 more min. Fig. 8B shows the time course averages for these stability tests. The first two measurements (at times 0 and 5 min) on each day are averaged to define the baseline for that day. All points are plotted as a percent change from baseline averaged across days.

**Figure 9:**
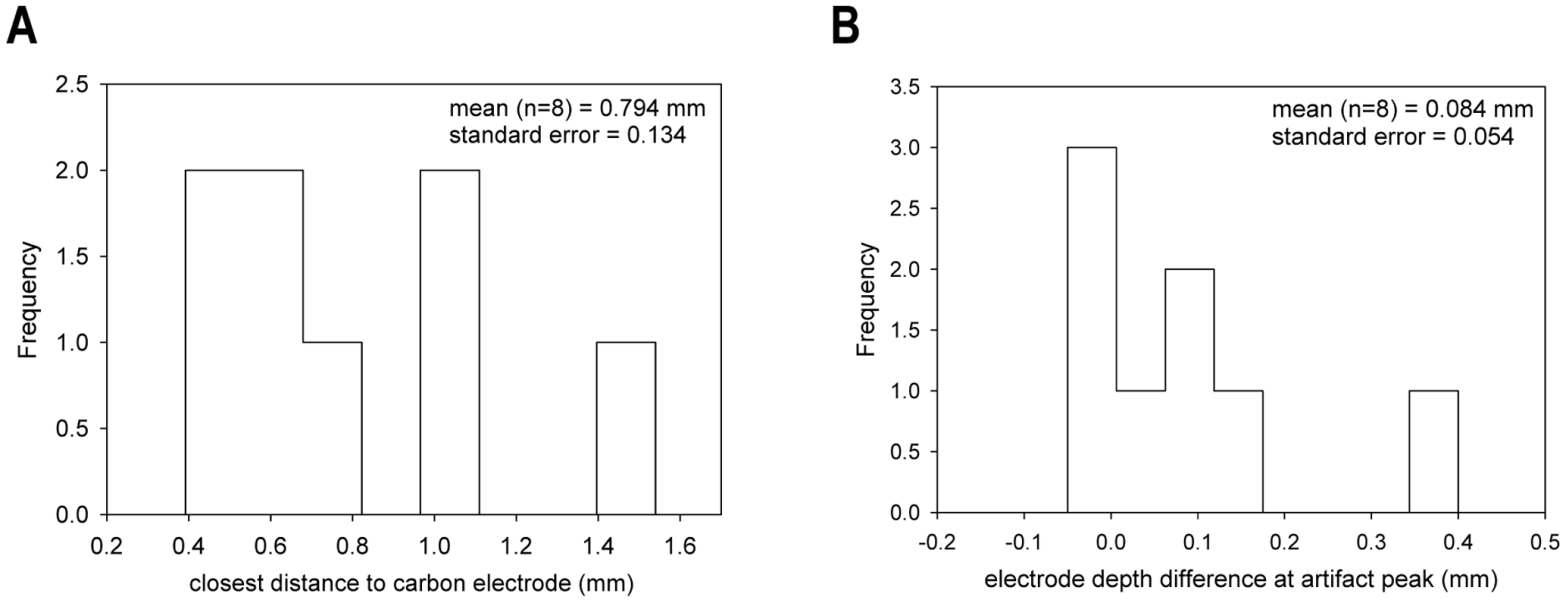
Accuracy of electrode positioning. Errors in drive position along different dimensions as estimated by using the stimulus artifact. (A) Distribution of nearest points between electrodes when using grid holes with 1 mm separation. (B) Distribution of differences between depth of peak artifact and point where both electrodes have the same depth reading. Positive values indicate the stimulation electrode was more superficial than expected.

Based upon depth measurements, the clearest examples of recorded DA did not seem to occur when the two electrodes were adjacent. When the electrodes were at their closest point, the DA oxidation potential was often obscured by other signals on the voltammogram (not shown), presumably related to shifts in pH or Ca^2+^ ions [18], [21]. However, when the electrodes were farther apart (>2 mm), we were not able to evoke a DA response. To verify these distance observations, we examined the accuracy of electrode position measurements by artifact analysis (see Appendix S2). Measured errors in electrode position were within ±0.5 mm along the XY plane of the microdrive (Fig. 9A). Relative depth readings were accurate and stimulation electrode positions were systematically a little more superficial than expected (Fig. 9B). We confirm that a separation of 200–500 µm was beneficial for observing DA.

### Juice Reward

**Figure 10:**
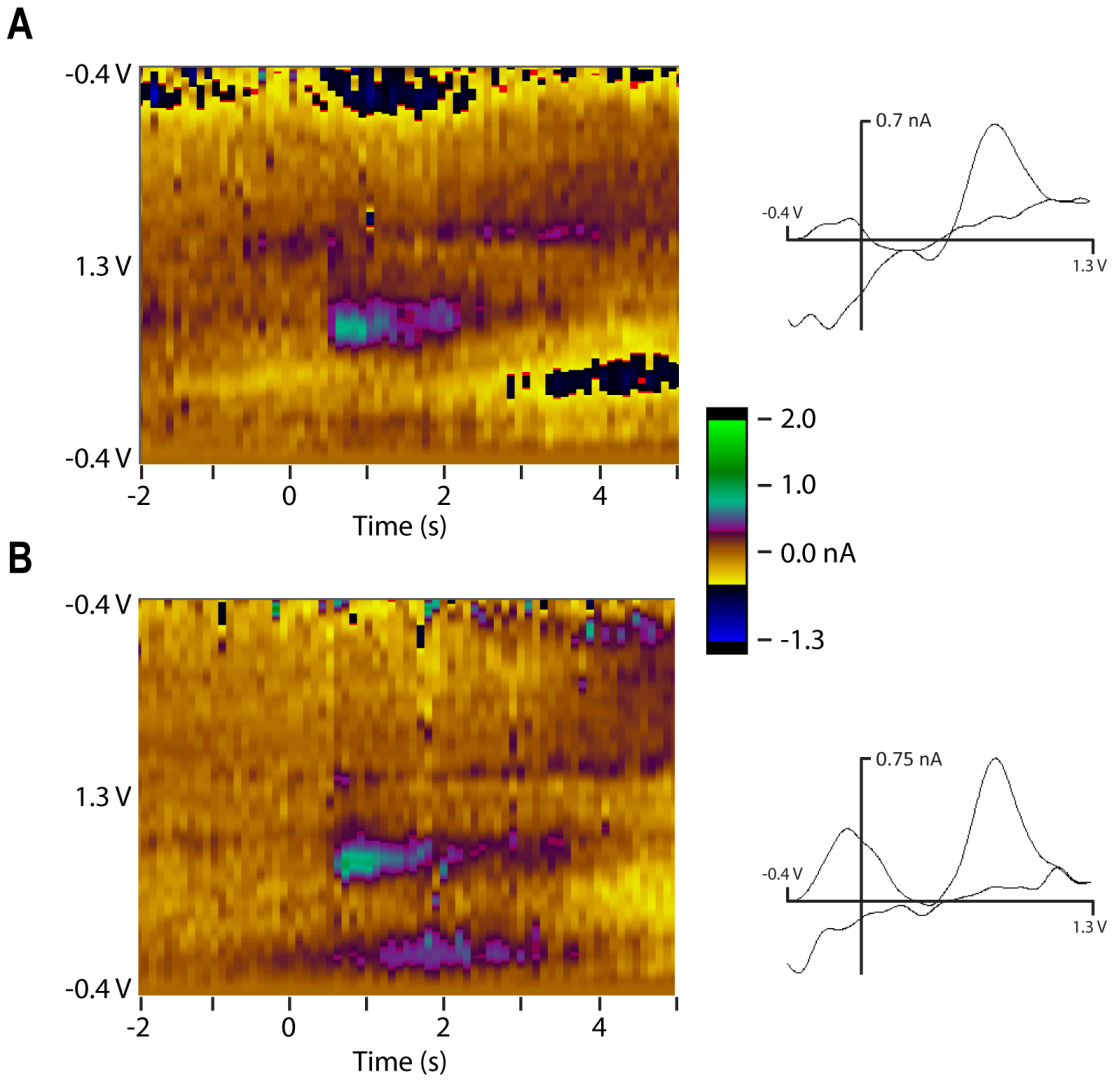
Reward related DA release in the ventral striatum. Triplets of juice rewards were delivered unexpectedly to monkey B, repeated 15 times over the course of 98(relative to ear bar zero, see [25]). Small, but constant dopamine responses occurred with every triplet. Example color plot and accompanying cyclic voltammograms for unexpected juice triplets delivered (A) 69.5 min after the first juice delivery and (B) 30 s later. Dopamine oxidation peaks are about 0.82 V.

In monkeys and rodents, DA neurons increase their firing rates, or DA is released when the animal receives an unexpected rewarding stimulus [41], [42], which can depend upon the behavioral state [43]. To examine responses to unexpected rewards, apple juice was delivered with a precision pressure driven liquid delivery system [44]. We manually delivered three ∼0.5 ml squirts of juice in rapid succession to the monkey. We were able to evoke reward-related DA during 4 of 10 experiments over a 7-week period. In two experiments, DA was evident after every reward trio (15/15 over 98 min and 7/7 over 28 min). In one experiment, DA was evoked only about half the time (6/13 over 177 min). In the last case, DA was evoked only once (1/4 over 70 min). Fig. 10 shows two example responses from one experiment. The responses were relatively small (<1 nA) compared to stimulation-evoked responses, but occurred with all 15 trios of rewards over 98 min. The response amplitudes are consistent with those observed in rodent studies.

## Discussion

We have demonstrated that FSCV can be used to measure transient DA release in monkey striatum using three different methods: recording DA responses time-locked with electrical stimulation in VTA/SNc (Fig. 5), by recording DA responses time-locked with local electrical stimulation in the striatum (Fig. 8), and by recording DA responses time-locked to unexpected rewards (Fig. 10). As expected from the rat literature (e.g., [31]), the responses to behaviorally evoked DA in the monkey were about an order of magnitude smaller than electrically evoked responses. Electrical stimulation helps locate DA rich sites in the striatum, providing a method to position an electrode prior to behavioral experiments [32]. Stimulation is also an aid for observing some pharmacological manipulations since it provides a robust DA signal. Both targets of electrical stimulation are helpful for validating our method, but each has its own benefits. Local stimulation was a more reliable way to achieve putative evoked DA release. Reliability is important for pharmacological manipulations that will further validate that DA is the source of the signal being measured. For now, VTA/SNc stimulation provides the best demonstration that DA is the chemical species being measured, because its projection to the striatum comes specifically from DA-containing neurons.

**Figure 11:**
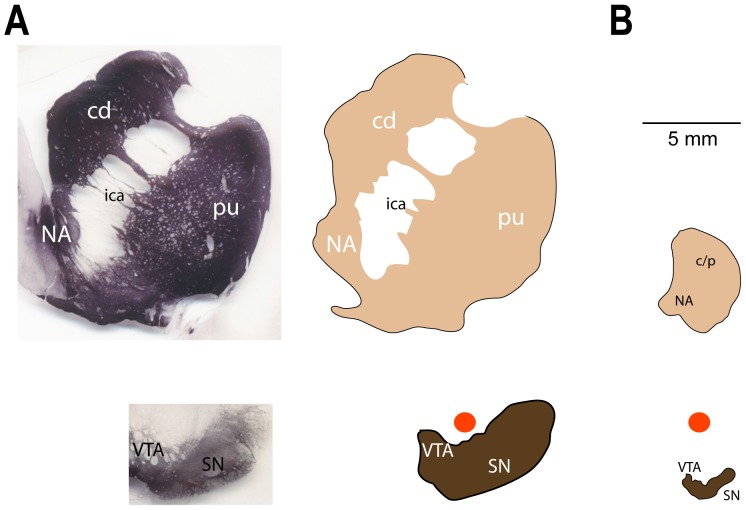
Monkey and rat striatum and VTA/SNc drawn to the same scale. Outlines of representative frontal sections from monkey and rat, striatum and VTA/SN. (A) Photomicrographs (left) of monkey tyrosine hydroxylase (TH) stained sections sized to the scale bar dimensions in B. Territory of monkey (right) striatum and VTA/SN based upon TH sections. (B) Outlines of dopamine neuron cell bodies and terminals in the rat VTA/SN (bottom, plate 43) and striatum (top, plate 17) from Paxinos and Watson atlas [50] estimated using the AChE stains. Circles above VTA/SN are each 1 mm diameter. Approximate area ratios (monkey:rat): striatum including ica = 8.6∶1, VTA/SN = 11.2∶1. NA - nucleus accumbens, cd - caudate, pu - putamen, ica - internal capsule, c/p - combined caudate/putamen.

Compared to rats [11], [31], [45], finding effective stimulation sites in monkeys VTA/SNc is more difficult. We found active sites of DA release paired with VTA/SNc sites 10–25% of the time in our two monkeys. This relatively low yield is best understood by comparing the anatomical differences between species. Fig. 11 charts the relative sizes of striatum and VTA/SNc in the two species on a common scale by comparing representative coronal sections. DA cell bodies are distributed throughout the VTA/SNc in both the rat [23] and monkey [3]. In the rat, the VTA/SNc is readily activated with 100–350 µA of current (e.g., [16], [31], [45]). Although bipolar electrodes are most commonly used, current spread to (DA-containing) cell bodies near the electrode tips will be comparable to monopolar stimulation when balanced pulses are used [30], [46]. More importantly, the whole of the rat VTA/SNc can be activated at these currents because the DA neurons are all within 2 mm of each other (Fig. 11B, [47]). In the monkey, DA neurons are distributed over the entire 7–8 mm long axis of the VTA/SNc (Fig. 11A, [3]). The current strengths used in rats can activate only a fraction of the DA-containing neurons in monkeys. Larger currents (e.g., [23]) spread outside the VTA/SNc before an appreciable increase in the number of activated DA neurons owing to the elongated shape of the VTA/SNc (Fig. 11A, bottom) and the high input resistances of the lightly myelinated DA fibers. This current spread outside VTA/SNc evoked movements from the RN and/or cerebral peduncle (Fig. 7). Movements interfered with recordings and even higher currents would likely be noxious to the animal. Thus, only a fraction of the DA neurons can be excited during any one penetration into the VTA/SNc using the angle of penetration we employed. Concomitantly, only a restricted region of the striatum will experience evoked DA release. Searching for the region of striatum experiencing the DA release is hampered by the large territory of the monkey striatum (Fig. 11A, top), especially compared to the rat striatum (Fig. 11B, top). In these illustrative sections, the monkey striatum is approximately 8.6 times the size of the rat striatum. The actual search territory is, of course, volumetric across the anterior-posterior dimension.

We chose to use new voltammetry electrodes and make new penetrations with our microdrive each recording day rather than using long-term recording from a single location [27]. This allowed us to search broadly for sites where DA release could be evoked by the stimulation electrode, to investigate the distribution of active release sites in the large primate brain and to evaluate the condition of the electrode each day after moving it through cm of brain. The disadvantage of this approach is the cumulative damage incurred by daily tracks. Future work would benefit from a low-profile microdrive that could leave the electrode implanted for weeks, permit electrode movement and allow insertion of a new electrode from time-to-time.

Our microdrive system did not reach into the center of the VTA (Fig. 6), because our chamber was mounted in the horizontal plane. Reaching the center of VTA would have required breaching the superior sagittal sinus with a guide cannula, a risky procedure. As just discussed, although electrical stimulation centered in the VTA will activate the vast majority of midbrain dopamine neurons in the rat, in monkeys it has a restricted projection, primarily to the nucleus accumbens [3]. Using a moveable electrode in monkeys offers an opportunity to explore micro-domains of DA release that may be governed by different regions of the VTA/SNc. During our stimulation experiments, we explored nucleus accumbens less than other parts of striatum because each penetration into that region was accompanied by damage to the more dorsal striatum, and because the center of VTA was difficult to reach. Exploration of nucleus accumbens in concert with VTA stimulation would likely yield additional DA release sites.

There are a number of ways to improve the yield of FSCV DA recordings during VTA/SNc stimulation in monkeys. The evoked DA release shown in Fig. 5A was found in the ventral striatum during stimulation of the ventromedial aspect of the SNc, in rough correspondence with the general topographic organization described by Haber [3]. A detailed map of the VTA/SNc projection could guide electrode placement. This might be accomplished by more fine-grained analysis of existing tracer data or through electrical stimulation mapping with fMRI. Even without additional information, it would be possible to reach more of the VTA and striatum, including medial portions, with a more lateral approach of the stimulating electrode. In this arrangement, a chamber could be tilted along the long axis of the VTA/SNc. A different approach was used by Shon et al. [48] in another large mammal. They evoked DA release over a wide area of the striatum using high levels of stimulation centered on the subthalamic nucleus. To activate such a large area, the animal had to be anesthetized and movements inhibited with additional muscle relaxants.

We propose an alternative strategy for increasing the number of VTA/SNc stimulation-evoked DA responses in awake monkeys. The long axis of the monkey VTA/SNc is well suited for implantation of a passive linear array electrode (e.g., V-Probe, Plexon, Inc., Dallas, TX). Available contact sizes include a diameter of 40 µm, which yields a nearly ideal surface area for stimulation (1257 µm^2^). Using 32 contacts spaced 200 µm apart, most of the midbrain dopamine neuron cell bodies would be within 2 to 3 mm of a stimulation contact. Given a suitable switching circuit, electrical stimulation could be tested at each site with monopolar stimulation, or at each pair of sites with bipolar stimulation. The entire VTA/SNc could be activated simultaneously using continuous interleaved sampling, a methodology developed for auditory prosthetics [39]. In this scheme, only one contact receives a stimulation pulse at any given moment, but all contacts receive a complete stimulus train nearly contemporaneously. Readily available electrophysiology equipment can generate these stimuli and synchronize with voltammetry (e.g., AlphaLab SnR, Alpha-Omega USA, Inc., Alpharetta, GA).

Local stimulation was a more reliable method for locating sites of DA release in the monkey. The optimal position for the stimulation electrode was near the carbon fiber electrode, but at least a few hundred µm away. Stimulating either too close to the carbon fiber electrode or with too high currents caused large artifacts that interfered with DA recordings. These observations are consistent with the distances most commonly used for brain slices (e.g., [10],[40]). The greater ease of finding DA responses with local stimulation provides utility in multiple ways. Local stimulation can be used to test pharmacological manipulations, as others have done (e.g., [11]). Because local stimulation can produce a large DA signal, it can be used to locate local DA rich areas.

The present work lays the foundation for studying DA activity in awake, behaving monkeys, essential for understanding the influence of DA upon complex behavior. Non-human primates are critical to pre-clinical studies towards human voltammetry applications, e.g., voltammetric recordings in concert with deep brain stimulation [20], [49]. We have addressed key methodological challenges associated with navigating a larger brain in an awake primate model. We have shown that stimulating DA terminals directly, and the cell bodies in the VTA/SNc, can each evoke phasic DA release.

## Supporting Information

Appendix S1 **Microprocessor-based stimulation controller.** A microprocessor circuit was used to control electrical stimulation pulse timing. The device synchronizes with the FSCV clock signal to avoid interfering with voltammetry recordings. A best-effort heuristic algorithm interleaves stimulation pulses with FSCV cycles over a wide range of pulse rates and durations. One option overlaps the first pulse of a pulse train with a FSCV cycle to display the timing of that event as an artifact on the voltammogram.
(PDF)

Appendix S2 **Estimating the accuracy of electrode trajectories in the brain.** The distance between two electrodes in the brain can be estimated using stimulation artifacts as long as one electrode is movable. We describe the geometry, application and a simplified method to estimate distance.
(PDF)
