# Supplementary material for: Real-Time Dopamine Measurement in Awake Monkeys
Source: PLoS One. 2014 Jun 12;9(6):e98692. doi: 10.1371/journal.pone.0098692 (PMC4055617; doi:10.1371/journal.pone.0098692)
Supplement: Appendix S1 — Microprocessor-based stimulation controller. A microprocessor circuit was used to control electrical stimulation pulse timing. The device synchronizes with the FSCV clock signal to avoid interfering with voltammetry recordings. A best-effort heuristic algorithm interleaves stimulation pulses with FSCV cycles over a wide range of pulse rates and durations. One option overlaps the first pulse of a pulse train with a FSCV cycle to display the timing of that event as an artifact on the voltammogram. (PDF) [file pone.0098692.s001.pdf]

Appendix S1

Microprocessor-based stimulation controller.

The timing of electrical stimulation was controlled by a custom circuit called Stimulation and fMRI Synchronizer (SfS). This device uses a microprocessor-based controller (PIC32MX795F512L, Microchip Technology, Chandler, AZ) to manage the sequencing of electrical stimulation for FSCV experiments. The FSCV equipment (Section 2.7.1) provides a CV_CLOCK digital output that goes low for each FSCV scan (Fig. 1A, top trace). During that ~10 ms period, electrical stimulation can induce a current artifact into the voltammetry measurement. Stimulation pulses outside of that time period are not reflected in the voltammogram. The SfS can be programmed to provide timing signals for electrical stimulation in such a way as to avoid the scan periods (Fig. 4A, middle trace). The SfS uses heuristics to generate a pulse train with approximately uniform interpulse intervals. A best-effort algorithm is used to guarantee the number of pulses and the scan window avoidance scheme, but it permits small inaccuracies in the overall programmed pulse train duration. The SfS also offers an option to position the first pulse of a pulse train within the first voltammetry sweep. Because a DA response is too slow to appear within 5 ms of electrical stimulation, there is no adverse consequence to an artifact in the voltammogram on the first sweep associated with a stimulation pulse train. In fact, such an artifact is desirable. It provides a convenient visual marker to locate the start of electrical stimulation directly on the voltammogram. Fig. 1B shows the timing of such a scheme. The first stimulation pulse (leftmost pulse, center trace) overlaps with the first time the CV_CLOCK (upper trace) drops, but the subsequent stimulation pulses avoid all subsequent CV_CLOCK periods. This mode of the SfS, where the first scan is “marked” with a stimulus artifact and the subsequent pulse train pulses do not overlap with FSCV sweeps, was used most commonly.

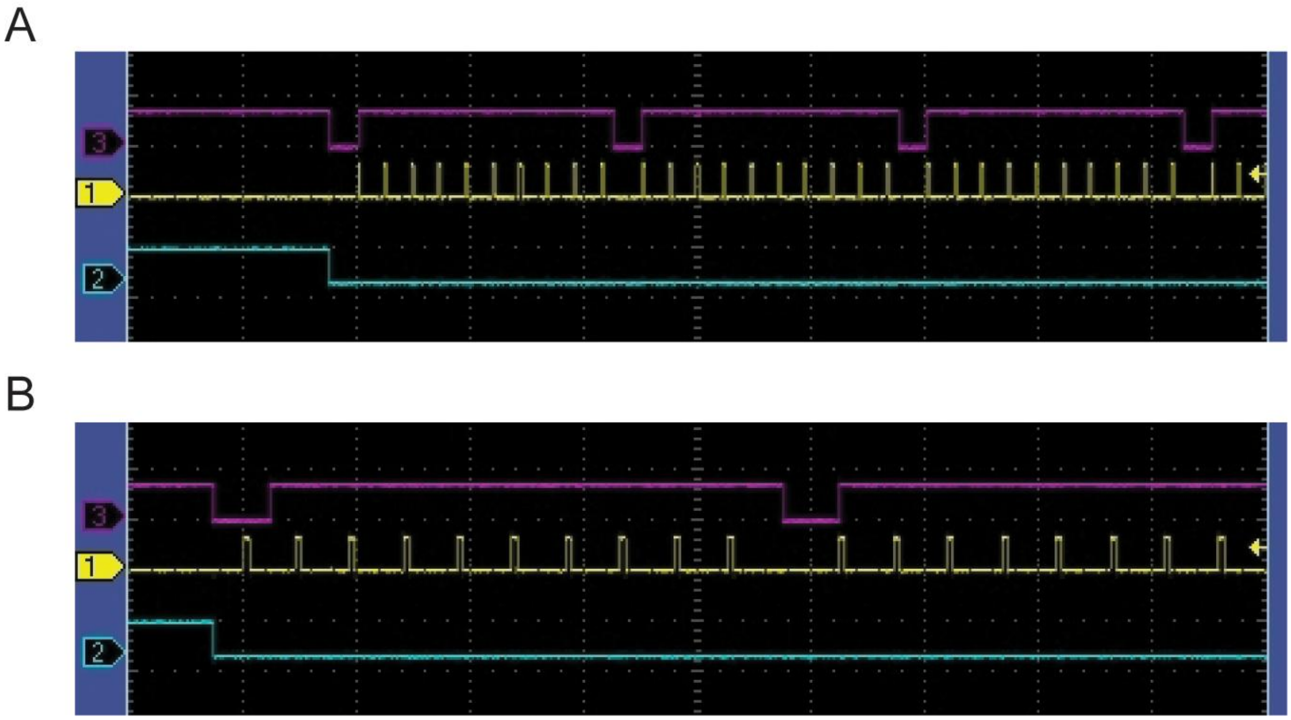

Fig. 1. Oscilloscope traces of signals managed by the Stimulation and fMRI Synchronizer (SfS)

In each panel, the top trace is a simulated CV_CLOCK signal; the second trace is the beginning series of synchronizing pulses that drive the stimulator; the third trace is the gate signal for shorting the stimulator output in between pulse trains. Simulated FSCV sweep is 10 ms followed by 90 ms period between sweeps. Stimulation at 100 PPS. (A) Stimulation synchronizing pulses are programmed to avoid all FSCV sweeps. Note gap after every 10th pulse to accommodate the CV cycle. (B) Stimulation synchronizing pulse of first sweep occurs in the middle of the sweep. Subsequent sweeps have the same characteristics as (A) above. The sweep speed of this trace is doubled to enlarge the CV cycle period.
