# Supplementary material for: Real-Time Dopamine Measurement in Awake Monkeys
Source: PLoS One. 2014 Jun 12;9(6):e98692. doi: 10.1371/journal.pone.0098692 (PMC4055617; doi:10.1371/journal.pone.0098692)
Supplement: Appendix S2 — Estimating the accuracy of electrode trajectories in the brain. The distance between two electrodes in the brain can be estimated using stimulation artifacts as long as one electrode is movable. We describe the geometry, application and a simplified method to estimate distance. (PDF) [file pone.0098692.s002.pdf]

Appendix S2

Estimating the accuracy of electrode trajectories in the brain: an example using a Tungsten stimulation electrode and a carbon fiber (CF) voltammetry electrode.

Geometry

The accuracy of electrode placement using a microdrive is often a concern. It would be valuable to have a simple method to estimate the precision and accuracy of electrode placement. In any two-electrode system, where one of the electrodes can be advanced along a relatively straight trajectory, it is possible to use microstimulation to estimate how close the advancing electrode approaches a fixed one. Repeating this measurement multiple times offers a statistical look at the precision and accuracy of the positioning system.

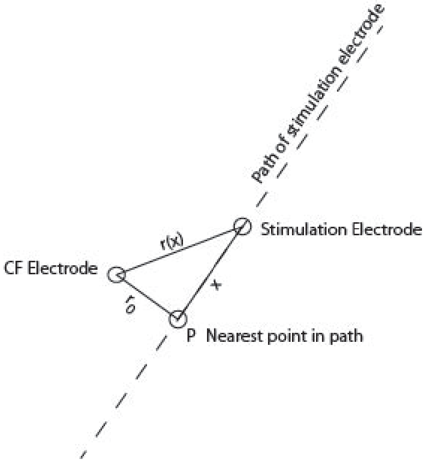

Fig. 1

We model a current-controlled stimulation electrode as a point source and a CF electrode as a small surface, both in a homogeneous volume conductor (Fig. 1). The CF electrode is held fixed. For the

purpose of the analysis, we model the stimulation electrode path with a straight line that has a nearest point at location P. Let,

$$r_0 = \textit{nearest distance between electrodes (CF to point} P\textit{)}$$

$$x = \textit{distance along path from point } P$$

From the Pythagorean Theorem:

$$r^2 = r_0^2 + x^2 \qquad (1)$$

Volume conduction

Stimulation current, i_s passes through a spherical surface of radius, r at the CF electrode. The proportion of current flowing through the surface area of the electrode is:

$$i = K_e \frac{i_s}{r^2} \qquad (2)$$

Where, K_e is a constant that reflects the effective surface of the electrode. The current is treated as a scalar quantity by assuming that the stimulation pulse is long enough to ignore reactance. Because the experiments are done at a fixed current, we define:

$$K = K_e i_s$$

From 2,

$$i = K \frac{1}{r^2} \qquad (3)$$

Combining 1 and 3

$$i = K\frac{1}{r_0^2 + x^2} \qquad (4)$$

`The maximum current, i_m will be recorded when the two electrodes are closest to each other.

At this point, P, $x = 0$. Evaluating (4) at $x = 0$, $i = i_m$, and solving for K

$$K = r_0^2 i_m \qquad (5)$$

Combining 4 and 5

$$i = \frac{r_0^2 i_m}{r_0^2 + x^2} \qquad (6)$$

We normalize the current by dividing by the maximum current.

$$i_N = \frac{i}{i_m} = \frac{r_0^2}{r_0^2 + x^2} \qquad (7)$$

Fig. 2 shows i_N for different values of r_0.

Note that at $x = r_0$,

$$i_N = \frac{r_0^2}{r_0^2 + r_0^2} = \frac{1}{2} \qquad (8)$$

Generalization

Although the example assumes a current-controlled stimulation source and a current monitor, voltage-controlled pulses and/or voltage monitoring (e.g., a system typically used for single unit recording) can

produce equivalent results. The only assumption is that the electrode impedances do not change during the measurement and the recording system is not saturated. From a practical standpoint, these assumptions are easily met by avoiding excessive stimulation currents.

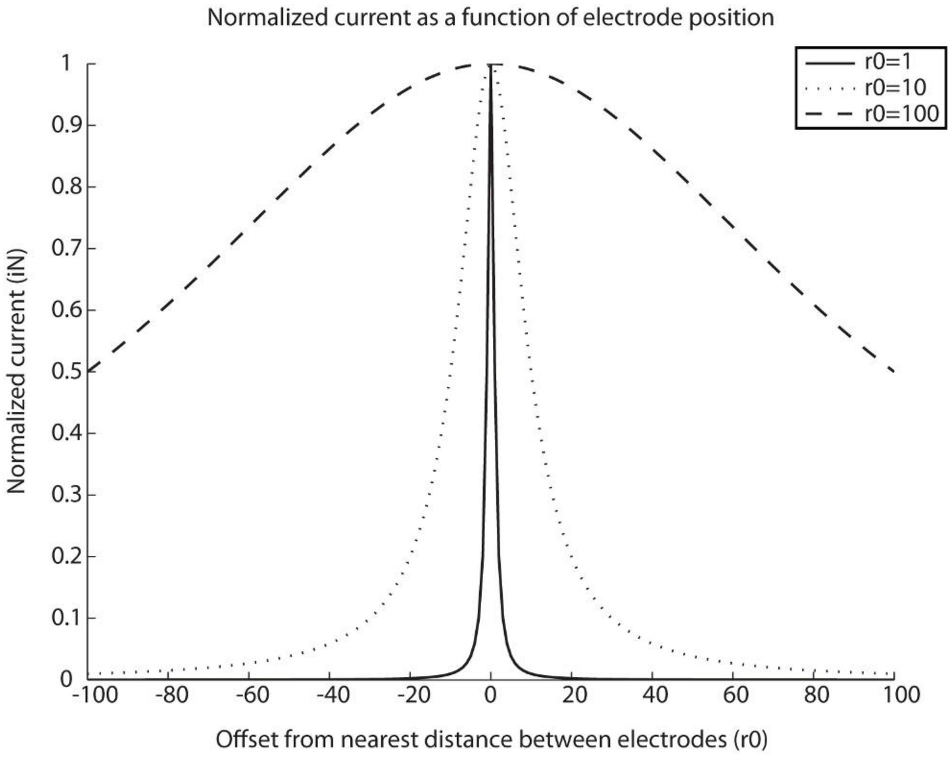

Fig. 2

Application

An estimate for r_0 starts by collecting samples of current versus electrode depth. Electrode depth is rescaled, setting the point of maximum current at $x = 0$. The values of current are normalized by dividing by the maximum current. The distance versus normalized current data are fitted to equation (7) and then the value of r_0 can be determined from the fitting parameters, or the value of x for $i_N = 0.5$ can be determined graphically. Repeated measures can be used to assess the overall reliability of electrode positioning. Given enough samples, and if we assume errors have a Gaussian distribution, the mean and standard deviation of the samples can be used to estimate the frequency of positioning error. Positioning error may accumulate with recording depth. In this case, it may be more useful to make measurements at multiple depths and translate position error into trajectory angle error. Measures of skewness may be effective tools for testing the straight-line assumption of electrode trajectory. Equation (8) offers a simple way to quickly asses the minimum distance between electrodes: find the maximum current and measure the distance from that point to the location where the evoked current drops by 50%. That distance is equal to r_0. Measuring the 50% point on either side of r_0 can also be a rapid assessment of trajectory linearity.
